# A phase III study comparing preservative-free latanoprost eye drop emulsion with preserved latanoprost in open-angle glaucoma or ocular hypertension

**DOI:** 10.1038/s41433-025-03646-z

**Published:** 2025-02-25

**Authors:** Christophe Baudouin, Ingeborg Stalmans, Rupert Bourne, Jose Manuel Larrosa, Stefanie Schmickler, Aleksey Seleznev, Francesco Oddone

**Affiliations:** 1 Hôpital National des Quinze-Vingts, IHU FOReSIGHT, INSERM-DGOS CIC 1423, Paris, France; 2 Institut de la Vision, IHU FOReSIGHT, Sorbonne University and Versailles Saint Quentin - Paris Saclay University, Paris, France; 3 Department of Ophthalmology, University Hospitals UZ Leuven, Leuven, Belgium; 4 Research Group Ophthalmology, Department of Neurosciences, Catholic University KU Leuven, Leuven, Belgium; 5 Glaucoma Service Lead & Director of Cambridge Eye Research Centre, Cambridge University Hospitals, Cambridge, UK; 6 Vision & Eye Research Institute, School of Medicine, Anglia Ruskin University, Cambridge, UK; 7 Hospital Universitario Miguel Server, University of Zaragoza, Zaragoza, Spain; 8 Augen-Zentrum-Nordwest, Ahaus, Germany; 9 Regional Budgetary Institution of Healthcare, Ivanovo Regional Clinical Hospital, Ivanovo, Russia; 10 Federal State Budgetary Educational Institution of Higher Education, Ivanovo State Medical Univeristy of the Ministry of Health of the Russian Federation, Ivanovo, Russia; 11 Glaucoma Unit, IRCCS-Fondazione Bietti, Roma, Italy; 12 Klinikum Klagenfurt am Woerthersee, Klagenfurt, Austria; 13 Department of Clinical Pharmacology, Medical University of Vienna, Vienna, Austria; 14 Ordination Dr. Georg Mossböck, Engelgasse 6, Graz, Austria; 15 University Hospital Leuven, Leuven, Belgium; 16 Eye Clinic of Tartu University Hospital, Tartu, Estonia; 17 Dr Kai Noor Silmakabinet OÜ, Katusepapi 6 Tallinn, Katusepapi 6 Tallinn, Tallinn, Estonia; 18 Eye Clinic of East Tallinn Central Hospital, Tallinn, Estonia; 19 Kuopio University Hospital and University of Eastern Finland, Kuopio, Finland; 20 Clinique Jules Verne, Nantes, France; 21 Hopitaux Universitaires Paris-Sud - Hopital Bicetre, Service d’Ophtalmologie, Paris, France; 22 CHU Bordeaux, Department of Ophthalmology, University of Bordeaux, INSERM, Bordeaux Population Health Research Center, team LEHA, Bordeaux, France; 23 Nantes Université, Centre hospitalier universitaire Nantes, Nantes, France; 24 Universitaetsmedizin der Johannes Gutenberg-Universität Mainz, Augenklinik und Poliklinik, Klinisches Studienzentrum der Augenklinik, Mainz, Germany; 25 Universitaetsklinikum Magdeburg A.oe.R, Magdeburg, Germany; 26 Universitaetsklinikum Hamburg-Eppendorf, Hamburg, Germany; 27 Augenzentrum Dr. Hamacher, Starnberg, Germany; 28 Augen-Zentrum Nordwest, Ahaus, Germany; 29 Universitaets Augenklinik Muenster, Muenster, Germany; 30 IRCCS Fondazione Bietti, Rome, Italy; 31 ASST Fatebenefratelli Sacco – P.O. L. Sacco-Clinica Oculistica, Milan, Italy; 32 Dipartimento di Scienze Cliniche e Medicina Traslazionale, Università di Roma Tor Vergata, Rome, Italy; 33 Azienda Ospedale Universita Padova – Clinica Oculistica, Padova, Italy; 34 Fondazione IRCCS Policlinico San Matteo, Pavia, Italy; 35 Department of Medicine - Ophthalmology, University of Udine, Piazzale Santa Maria della Misericordia, Udine, Italy; 36 Department of Ophthalmology, Riga East University Hospital Clinical Center Bikernieki, Riga, Latvia; 37 Pauls Stradins Clinical University Hospital, Riga Stradins University, Riga, Latvia; 38 Oftalmologijas Sabiedriba Grund Opt, Valmiera, Latvia; 39 Wojskowy Instytut Medyczny, Warsaw, Poland; 40 Szpital Sw. Rozy, Krakow, Poland; 41 Uniwersyteckie Centrum Kliniczne im Prof. K. Gibińskiego Śląskiego Uniwersytetu Medycznego w Katowicach, Katowice, Silesia Poland; 42 Klinika Okulistyki WNMK Śląskiego Uniwersytetu Medycznego w Katowicach, Katowice, Silesia Poland; 43 Retina Szpital Okulistyczny, Warsaw, Poland; 44 Federal State Autonomous Institution National Medical Research Center Inter-Industry Scientific Technical Complex “Eye Microsurgery” N.A. Academician S.N. Fyodorov” Of The Ministry Of Healthcare Of The Russian Federation Beskudnikovskiy blvd., Moscow, Russia; 45 Budget Healthcare Institution Of Omsk Region “Clinical Ophatlmology Hospital N.A. V.P.Vykhodtseva”, Omsk, Russia; 46 Federal State Autonomous Institution “National Medical Research Center “Inter-Industry Scientific Technical Complex “Eye Microsurgery” N.A. Academician S.N. Fyodorov” Of The Ministry Of Healthcare Of The Russian Federation, Saint- Petersburg Branch, Novosibirsk Branch, Novosibirsk, Russia; 47 Federal State Budgetary Educational Institution of Higher Education “Academician I.P. Pavlov First St. Petersburg State Medical University” of the Ministry of Healthcare of the Russian Federation, St. Petersburg, Russia; 48 Federal State Autonomous Institution “National Medical Research Center “Inter-Industry Scientific Technical Complex “Eye Microsurgery” N.A. Academician S.N. Fyodorov” Of The Ministry Of Healthcare Of The Russian Federation, St. Petersburg Branch, St. Petersburg, Russia; 49 Limited Liability Company “Eye Diseases Clinic”, Kazan, Russia; 50 Federal State Budget Educational Institution of Higher Education “Moscow State University of Medicine and Dentistry n.a. A. I. Yevdokimov” of the Ministry of Healthcare of the Russian Federation, Moscow, Russia; 51 Private Healthcare Institution “Clinical Hospital “RZhD- Medicina”, Saratov, Russia; 52 Federal State Autonomous Institution “National Medical Research Center “Inter-Industry Scientific Technical Complex “Eye Microsurgery” N.A. Academician S.N. Fyodorov” Of The Ministry Of Healthcare Of The Russian Federation, Cheboksary Branch, Pr. Traktorostroiteley, Cheboksary, Russia; 53 RC Medical LLC, Novosibirsk, Russia; 54 Severance Hospital, Yonsei University College of Medicine, Seoul, South Korea; 55 Seoul National University Hospital, Seoul, South Korea; 56 The Catholic University of Korea, Seoul St. Mary’s Hospital, Seoul, South Korea; 57 Hospital Universitario de Torrevieja, Torrevieja, Spain; 58 Instituto Oftalmológico Gómez-Ulla, Santiago de Compostela, Spain; 59 Hospital Universitario Miguel Servet, Zaragoza, Spain; 60 Hospital Clínico San Carlos, Madrid, Spain; 61 Hospital Clinic de Barcelona, Barcelona, Spain; 62 Clínica Universidad de Navarra, Pamplona, Spain; 63 Instituto Oftalmológica Fernandez-Vega, Oviedo, Spain; 64 Centro de Oftalmología Barraquer, Barcelona, Spain; 65 Institut Catala de Retina, Calle Ganduxer, Barcelona, Spain; 66 West Suffolk Hospital NHS Foundation Trust, Bury Saint Edmunds, UK; 67 Cambridge University Hospitals NHS Foundation Trust - Addenbrookes Hospital, Cambridge, UK; 68 Portsmouth Hospitals NHS Trust, Queen Alexandra Hospital, Portsmouth, UK; 69 Imperial College Healthcare NHS Trust, London, UK

**Keywords:** Eye diseases, Diseases

## Abstract

**Background/Objectives:**

To evaluate the efficacy and safety of preservative-free latanoprost eye drop emulsion in reducing intraocular pressure (IOP) versus preserved latanoprost in open-angle glaucoma (OAG) or ocular hypertension (OHT).

**Methods:**

A Phase III non-inferiority study randomised patients with OAG/OHT 1:1 to receive preservative-free latanoprost eye drop emulsion or preserved latanoprost. The primary efficacy endpoint was change from baseline in peak (9:00 A.M. ± 1 h) and trough (4:00 P.M. ± 1 h) IOP at Week 12 (non-inferiority margin: 95% confidence interval for treatment difference of ≤1.5 mmHg). Key secondary endpoints were change from baseline in corneal fluorescein staining (CFS) score and in ocular surface disease (OSD) average symptom score at Week 12 (in patients with baseline CFS ≥ 1 or OSD score > 0, respectively).

**Results:**

Non-inferiority criteria for IOP-lowering were met. Least square (LS) mean (standard error [SE]) IOP change from baseline with preservative-free latanoprost eye drop emulsion (N = 193) versus preserved latanoprost (N = 193) at Week 12 was −8.8 (0.3) mmHg versus −8.2 (0.3) mmHg at peak (difference: −0.6 mmHg; nominal p = 0.023); −8.6 (0.2) mmHg versus −8.1 (0.3) mmHg at trough (difference: −0.5 mmHg; p = 0.080). LS mean change in CFS (SE) was −0.7 (0.07) with preservative-free latanoprost eye drop emulsion and −0.4 (0.08) with preserved latanoprost (nominal p < 0.001). LS mean change in OSD symptom score was −0.3 (0.1) with preservative-free latanoprost eye drop emulsion and −0.2 (0.1) with preserved latanoprost (nominal p = 0.090).

**Conclusions:**

Preservative-free latanoprost eye drop emulsion demonstrated non-inferior IOP-lowering efficacy compared with preserved latanoprost, and improved signs and symptoms of OSD.

## Introduction

Glaucoma is a multifactorial and neurodegenerative group of heterogeneous diseases characterised by retinal ganglion cell apoptosis, leading to progressive and irreversible visual field loss [1]. Glaucoma management aims to promote the patient’s well-being and quality of life by minimising glaucomatous vision loss [1]. Therapy aims to reduce intraocular pressure (IOP; the only modifiable risk factor) to prevent further damage to the optic nerve and preserve visual function and quality of life [1, 2]. Prostaglandin analogues (PGAs), such as latanoprost, are the standard first-line treatment for open-angle glaucoma (OAG) [1].

Ocular surface disease (OSD) occurs more frequently in patients with glaucoma than in the general population, reported in 22–78% of patients depending on the specific test used, compared with 5–30% overall in the general population [3–5]. OSD refers to a group of conditions that disrupt the normal functioning of the ocular surface and homeostasis of the tear film [6, 7]. Clinical signs of OSD include inadequate tear quantity, an unstable tear film, and damage of the ocular surface epithelia. Symptoms include irritation, burning, dryness, foreign body sensation, fatigue, photophobia and fluctuating visual acuity [3].

The pathogenesis of OSD in patients with glaucoma is likely related to chronic inflammation, which may be caused by glaucoma itself, or worsened by topical glaucoma medications [8, 9]. OSD symptoms may lead to a lack of glaucoma treatment adherence [7, 10, 11], and OSD can decrease the effectiveness of IOP-lowering medication [8].

Effective management of ocular surface health is important for patients with glaucoma and may improve the outcomes of glaucoma treatment and quality of life [5, 12, 13]. Managing OSD can involve artificial tears and anti-inflammatory agents, but these approaches do not address the underlying causes of OSD and increase treatment complexity, which may further reduce glaucoma treatment adherence [5, 7, 14]. OSD remains a challenge for patients with glaucoma; there is an unmet need for anti-glaucoma eye drops that consider the ocular surface beyond preservative-free formulations [7].

Cationic emulsions are oil-in-water formulations that mimic a healthy tear film and promote a healthy ocular surface environment [15, 16], with demonstrated clinical efficacy in improving the ocular surface [17, 18]. Cationic emulsion formulations are effective drug delivery systems due to their positive charge interacting with the negatively charged ocular surface. This promotes spreading and adherence of the formulation on the ocular surface [19, 20]. Preservative-free latanoprost eye drop emulsion is a cationic emulsion formulation of latanoprost 0.005% [20]. Preclinical data indicate effective delivery of latanoprost to ocular tissues, IOP-lowering, and restoring a diseased tear film [21, 22].

Preservative-free latanoprost eye drop emulsion has demonstrated promising clinical activity for IOP control, and improved OSD signs and symptoms in two Phase II studies [23, 24]. A Phase III randomised study was conducted to assess efficacy and safety of preservative-free latanoprost eye drop emulsion in reducing IOP versus preserved latanoprost in patients with OAG or OHT. Change from baseline in corneal fluorescein staining (CFS) score and OSD symptom score were assessed as key secondary endpoints.

## Materials and methods

### Study design

A Phase III, prospective, parallel, multinational, multicentre, double-masked, randomised, active-controlled, non-inferiority study (EudraCT: 2017-004262-95) was conducted to compare efficacy and safety of preservative-free latanoprost eye drop emulsion (preservative-free cationic emulsion formulation of latanoprost 0.005% [Santen Oy, Catiolanze®]) with preserved latanoprost (latanoprost 0.005% eye drop solution [Pfizer, Xalatan®]) in patients with OAG or ocular hypertension (OHT) over a 12-week period (Period 1), followed by a 12-month open-label period of preservative-free latanoprost eye drop emulsion treatment. A subset of patients who attended the Week 12 visit, and agreed to participate, continued preservative-free latanoprost eye drop emulsion treatment or switched to preservative-free latanoprost eye drop emulsion. The purpose of the open-label extension was to evaluate the long-term safety, tolerance, and efficacy of preservative-free latanoprost eye drop emulsion (Supplementary Fig. 1). Here, we report results from the 12-week non-inferiority period.

The study was conducted in 47 sites across Austria, Belgium, Estonia, Finland, France, Germany, Italy, Latvia, Poland, Spain, United Kingdom, Russia and South Korea.

The final protocol, its amendments, Informed Consent Form, relevant supporting information and patient recruitment information were approved by an Independent Ethics Committee and/or Institutional Review Board prior to study initiation. The study was conducted in accordance with International Council for Harmonisation of Good Clinical Practice. Written informed consent was obtained from all patients. Preserved latanoprost was requested as a study reference drug by the European Medicines Agency.

### Patient population

Male and female patients with OAG or OHT aged ≥18 years under monotherapy glaucoma treatment, with a stable visual field (6 months), and a post-washout IOP of ≥22 mmHg (≥1 eye) and ≤32 mmHg in each eye were included in the study. Key exclusion criteria included current glaucoma treatment with a fixed-dose combination or >1 therapy in either eye, or with an oral drug (within 6 months), corneal abnormalities, significant visual field loss (absolute defect in the 10° central point or mean deviation worse than −12 decibels), optic nerve abnormality (as determined by ophthalmoscopy in either eye), optic neuropathy changes (e.g., increase in cupping since the last examination or optic nerve haemorrhage in either eye), severe blepharitis and/or meibomian gland dysfunction (MGD), ocular infection or severe dry eye disease (DED) (CFS grade 4, modified Oxford Scale), current/anticipated steroid use (oral/topical/depot), intraocular surgery (within 6 months), refractive surgery (within 1 year), any history of filtering surgery, and pregnancy or breastfeeding.

### Treatment

At the screening visit, a washout phase of ≥5 days to 4 weeks (determined by the IOP-lowering medication used) was conducted. This lasted 4 weeks for prostaglandin analogues, ≥3 to ≤4 weeks for topical beta blockers, ≥5 days to ≤4 weeks for topical carbonic anhydrase inhibitors, and ≥2 weeks to ≤4 weeks for all other IOP-lowering medication. During the washout phase, patients received brinzolamide twice daily; brinzolamide administration was stopped 5 days before randomisation. Patients were randomised 1:1 to receive either preservative-free latanoprost eye drop emulsion (preservative-free cationic emulsion formulation of latanoprost 0.005%) or preserved latanoprost (latanoprost 0.005% eye drop solution) once daily at 9:00PM.

### Endpoints

The primary efficacy endpoint was the change from baseline in peak (9:00 A.M. ±1 h) and trough (4:00 P.M. ±1 h) IOP at Week 12. The non-inferiority margin was ≤1.5 mmHg 95% confidence interval (CI) for between-arm treatment difference.

Key secondary endpoints were changed from baseline in CFS score at Week 12 in patients with baseline CFS score  ≥ 1 and change from baseline in OSD symptom score (average of 3 symptoms: dry eye sensation, blurred/poor vision, and burning/stinging/itching) at Week 12 in patients with baseline average symptom score > 0.

Other pre-specified secondary endpoints were change from baseline in IOP at Week 4 peak and trough, change from baseline in mean diurnal IOP (post-washout baseline IOP) at Week 12, IOP reduction of ≥20%, ≥25% and ≥30% from baseline at Week 12 peak and trough, IOP ≤ 18 mmHg at Week 12 peak and trough, Patient Global Rating of Treatment at Week 12, quality of life, tear-film breakup time (TFBUT), conjunctival hyperaemia and slit lamp examination (MGD, conjunctiva chemosis, and lid and tear film debris) at Weeks 4 and 12, and safety.

### Efficacy and safety assessments

IOP was measured using calibrated manual Goldmann applanation tonometry. Measurement was performed by the same investigator and same recorder throughout the study. The modified Oxford scale was used to grade CFS, and measures involved examination of the eye at a slip lamp using a yellow barrier filter and cobalt blue illumination to enhance visibility of staining. OSD symptoms were graded as 0 (absent), 1 (mild), 2 (moderate), 3 (severe) or 4 (very severe). To assess Patient Global Rating of Treatment, patients were asked to select one of the following four choices: unsatisfactory, not very satisfactory, satisfactory, or very satisfactory. At each visit, TFBUT was measured twice in each eye: 2 μL of non-preserved 2% sodium fluorescein was instilled onto the bulbar conjunctiva of each eye without inducing reflex tearing by using a micro-pipette. The TFBUT value was the average of two or three measurements. Conjunctival hyperaemia was measured at Weeks 4 and 12 using a slit lamp and scored using the photographic scale derived from McMonnies scale (1–6). Safety assessment included reported adverse events (AEs) and treatment-related AEs, best-corrected distance visual acuity and slit-lamp examination of both eyes. Each AE was classified into a System Organ Classification and coded to a preferred term and a lowest level term using Medical Dictionary for Regulatory Activities Version 23.1.

### Statistics: randomisation and stratification

The planned sample size was determined assuming a mean difference in IOP change from baseline of 0 mmHg and a common standard deviation (SD) of 4.3 mmHg in the peak or trough IOPs, respectively, for the comparison between the preservative-free latanoprost eye drop emulsion and the preserved latanoprost arms. Three hundred and eighty patients (190 per treatment arm) would provide 90% power to demonstrate non-inferiority of preservative-free latanoprost eye drop emulsion compared with preserved latanoprost (one-sided α = 0.025) for a non-inferiority margin at 95% CI for treatment difference of ≤1.5 mmHg, assuming a 10% dropout rate. The difference in change from baseline in peak and trough IOP between two treatment arms at Week 12 was compared using a mixed-effects model for repeated measures (MMRM). A separate MMRM included treatment, visit and treatment-by-visit interaction as fixed effects, and baseline IOP at peak or trough and country as covariates. Within-patient errors were modelled using an unstructured covariance matrix. To control for overall Type 1 error rate associated with the multiple comparisons on the primary and key secondary endpoints at the 0.05 level (two-sided), a hierarchical testing strategy was employed. For the primary comparison, the difference between the two treatment groups in change from baseline in both peak and trough IOP at Week 12 9 A.M. and 4 P.M. had to meet the non-inferiority criterion that the upper limit of the one-sided 97.5% CI was ≤1.5 mmHg. If this was achieved, statistical testing for the key secondary endpoints was conducted sequentially according to hierarchical fixed sequence procedure. Regarding CFS at Week 12, if the hypothesis was rejected at 0.05 significance level (two-sided), OSD measure at Week 12 was tested. In addition to overall Type 1 error control between primary and secondary endpoints, 95% CIs and nominal p-values (non-adjusted) are reported for all other statistical tests. Statistical analyses were performed using the SAS® (Statistical Analysis Software) software version 9.4. Randomisation was stratified according to the CFS score of the study eye at baseline (CFS ≤ 1 versus CFS ≥ 2, modified Oxford scale). Efficacy was assessed in the full analysis set (FAS), consisting of all randomised patients who received at least one dose of the study medication, and provided at least one post-baseline IOP measurement at peak and trough timepoints. Sensitivity analysis of the primary efficacy endpoint was performed in the per-protocol population (all patients of the FAS without any of the major protocol deviations that could affect the primary efficacy endpoint; see Supplementary Statistical Information for further details). The safety population comprised all patients who received at least one dose of the study medication.

## Results

### Patients

Of 488 patients screened, 386 were randomised and received at least one dose of study medication (safety population; n = 193 in the preservative-free latanoprost eye drop emulsion arm and n = 193 in the preserved latanoprost arm) (Supplementary Fig. 2). One hundred and ninety-two patients in each treatment arm had at least one post-baseline IOP measurement at peak and trough times and were included in efficacy analysis (FAS). In total, 380 patients completed 12 weeks of treatment and six patients discontinued the study prematurely. Baseline demographics and disease characteristics were well-balanced in both treatment arms (Table 1). The mean (SD) age was 63.1 (11.2) years and 61.5% (n = 236) were female. The mean (SD) baseline diurnal IOP was 24.1 (1.8) mmHg. Overall, 72.8% (n = 279) were treated with PGA monotherapy prior to baseline. At baseline, 45.3% of patients (n = 174) had CFS ≥ 1, 67.5% (n = 259) had TFBUT ≤ 10 s, 55.5% (n = 213) had OSD symptom score > 0, and 30.5% (n = 117) had both CFS ≥ 1 and OSD symptom score > 0 (Supplementary Fig. 3).

**Table 1:** Baseline characteristics of patients included in the efficacy analysis.

Characteristic	Preservative-free latanoprost eye drop emulsion (N = 192)	Preserved latanoprost (N = 192)	Overall (N = 384)
Age, mean (SD)	62.3 (12.1)	63.9 (10.1)	63.1 (11.2)
Sex, n (%)			
Male	72 (37.5)	76 (39.6)	148 (38.5)
Female	120 (62.5)	116 (60.4)	236 (61.5)
Ethnicity, n (%)			
White	184 (95.8)	186 (96.9)	370 (96.4)
Asian	4 (2.1)	4 (2.1)	8 (2.1)
Other	4 (2.1)	2 (1.0)	6 (1.6)
Primary diagnosis, n (%)			
POAG	141 (73.4)	150 (78.1)	291 (75.8)
OHT	45 (23.4)	36 (18.8)	81 (21.1)
PEX	4 (2.1)	4 (2.1)	8 (2.1)
Pigmentary glaucoma	2 (1.0)	2 (1.0)	4 (1.0)
Time since diagnosis (year)			
Mean (SD)	5.9 (5.3)	6.1 (5.3)	6.0 (5.3)
Median (range)	4.0 (0–31)	4.5 (0–29)	4.0 (0–31)
Baseline IOP-lowering medication^a^, n (%)			
PGAs	142 (74.0)	137 (71.7)	279 (72.8)
CAIs	25 (13.0)	27 (14.1)	52 (13.6)
Beta blocking agents	15 (7.8)	21 (11.0)	36 (9.4)
Diurnal IOP (mmHg)			
Mean (SD)	24.1 (1.6)	24.2 (2.0)	24.1 (1.8)
Median (range)	23.8 (22.0–30.0)	23.6 (22.0–32.0)	23.8 (22.0–32.0)
CFS score			
Mean (SD)	0.7 (0.7)	0.7 (0.7)	0.7 (0.7)
Median (range)	0.5 (0.0–3.0)	0.5 (0.0–3.0)	0.5 (0.0–3.0)
CFS score^b^ (modified Oxford scale), n (%)			
0	58 (30.2)	60 (31.3)	118 (30.7)
0.5	49 (25.5)	43 (22.4)	92 (24.0)
1	56 (29.2)	62 (32.3)	118 (30.7)
2	27 (14.1)	24 (12.5)	51 (13.3)
3	2 (1.0)	3 (1.6)	5 (1.3)
TFBUT ≤10 s, n (%)	123 (64.1)	136 (70.8)	259 (67.4)
OSD symptoms, n (%)			
Score > 0	105 (54.7)	108 (56.3)	213 (55.5)

*CAI* carbonic anhydrase inhibitor, *CFS* corneal fluorescein staining, *IOP* intraocular pressure, *OHT* ocular hypertension, *OSD* ocular surface disease, *PEX* pseudo-exfoliative glaucoma, *PGA* prostaglandin analogue, *POAG* primary open-angle glaucoma, *SD* standard deviation, *TFBUT* tear-film breakup time.

^a^Other medications included sympathomimetics and combinations of the classes listed.

^b^There were no patients with CFS score 4 or 5.

No statistical testing for between treatment group differences was performed.

### IOP-lowering

**Fig. 1:**
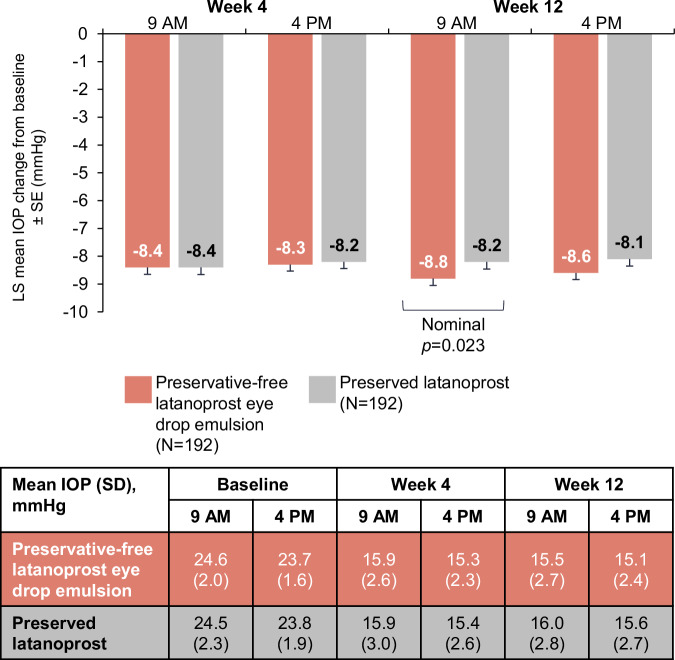
Mean IOP change from baseline in the efficacy population. IOP intraocular pressure, LS least square, SD standard deviation, SE standard error.

At baseline in the FAS, the mean IOP (SD) was 24.6 (2.0) mmHg at peak and 23.7 (1.6) mmHg at trough in the preservative-free latanoprost eye drop emulsion arm, and 24.5 (2.3) mmHg and 23.8 (1.9) mmHg in the preserved latanoprost arm, respectively. In the preservative-free latanoprost eye drop emulsion arm at Week 12 peak, least square (LS) mean (standard error [SE]) IOP change from baseline was −8.8 (0.25) mmHg, with a mean (SD) IOP of 15.5 (2.7) mmHg. At trough, the LS mean (SE) IOP change was −8.6 (0.2) mmHg, with a mean (SD) IOP of 15.1 (2.4) mmHg (Fig. 1). In the preserved latanoprost arm at Week 12 peak, LS mean (SE) IOP change from baseline was −8.2 (0.3) mmHg, with a mean (SD) IOP of 16.0 (2.8) mmHg. At trough, the LS mean (SE) IOP change was −8.1 (0.3) mmHg, with a mean (SD) IOP of 15.6 (2.7) mmHg. The pre-specified non-inferiority criteria of the upper limit of the 95% CI (≤1.5 mmHg) was achieved for both peak and trough measurements, with a treatment difference of −0.6 mmHg (95% CI: −1.2 to −0.1) and −0.5 mmHg (95% CI: −1.0 to 0.1), respectively. Preservative-free latanoprost eye drop emulsion demonstrated greater IOP reduction compared with preserved latanoprost was observed at Week 12 peak (nominal p = 0.023) but not at Week 12 trough (nominal p = 0.080).

Sensitivity analysis conducted on the per-protocol population was consistent with the main analysis, with non-inferiority criterion achieved for both peak and trough measurements; the upper limit of the one-sided 95% CI was ≤1.5 mmHg at both timepoints at Week 12. The difference in LS means (two-sided 95% CI) between preservative-free latanoprost eye drop emulsion and the preserved latanoprost was −0.6 (95% CI −1.1 to −0.0) at the peak timepoint, and −0.4 (95% CI −0.9 to 0.1) at the trough timepoint.

**Fig. 2:**
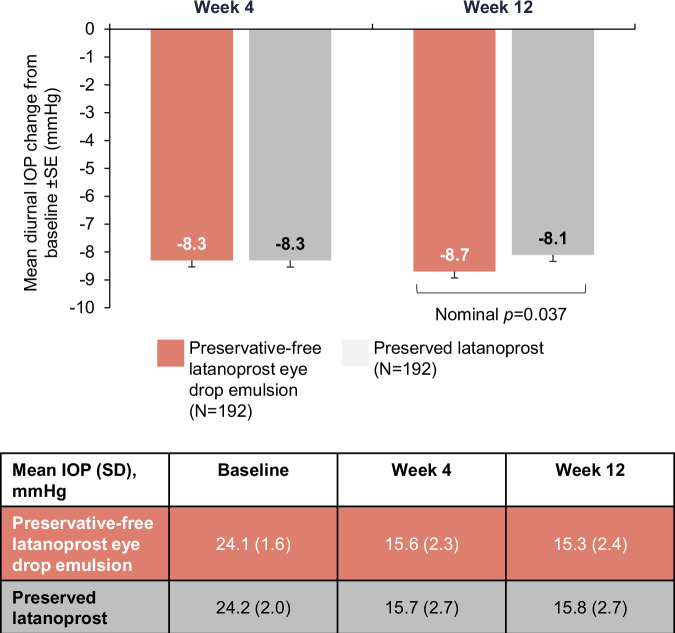
Mean diurnal IOP change at Week 12 in the efficacy population. IOP intraocular pressure, SD standard deviation, SE standard error.

Week 12 post hoc analyses show the LS mean diurnal IOP (SE) reduction was numerically greater with preservative-free latanoprost eye drop emulsion (−8.7 [0.2] mmHg) compared with preserved latanoprost (−8.1 [0.3] mmHg) and the treatment difference of −0.5 (0.3) mmHg favoured preservative-free latanoprost eye drop emulsion (nominal p = 0.037; Fig. 2). At Week 12 peak, the proportion of patients with decreases in IOP of 20% and 25% were similar in both arms (Supplementary Fig. 4). The proportion of patients with an IOP reduction of ≥30% was greater in the preservative-free latanoprost eye drop emulsion arm (74.5%) compared with the preserved latanoprost arm (64.0%; nominal p = 0.028). At Week 12 trough, there were more patients in the preservative-free latanoprost eye drop arm with an IOP reduction to ≤18 mmHg compared with the preserved latanoprost arm (87.6% vs 79.3%, respectively [nominal p = 0.029]).

### OSD improvement

**Fig. 3:**
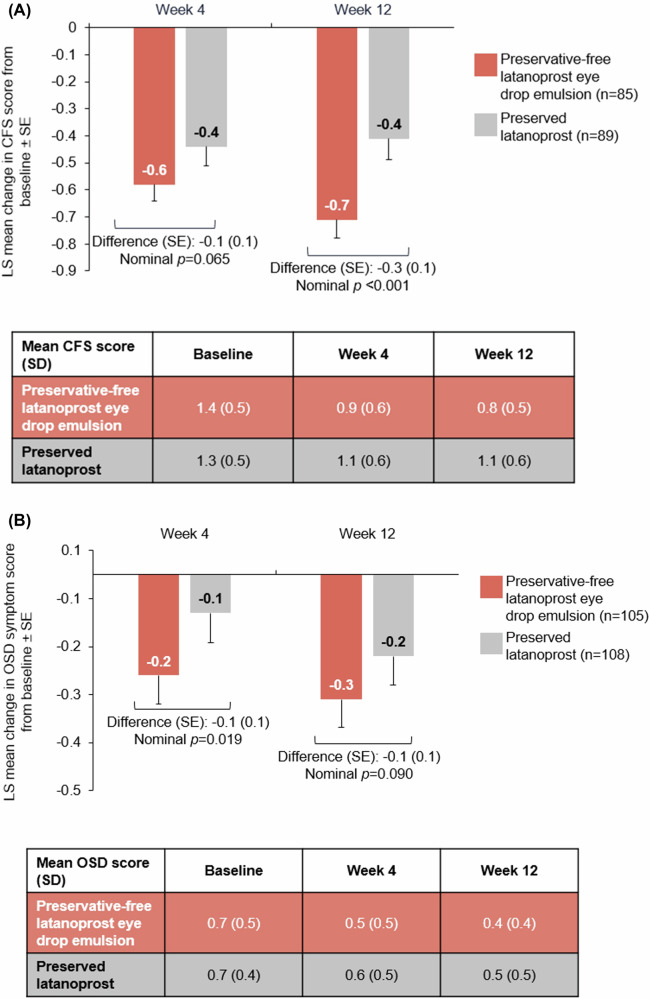
Mean change from baseline in CFS and OSD scores at Weeks 4 and 12 in patients with baseline CFS ≥1 or OSD score>0. Mean change in CFS (**A**) and OSD (**B**) scores in patients with baseline CFS ≥ 1 or OSD score >0 at Weeks 4 and 12. CFS corneal fluorescein staining, LS least square, OSD ocular surface disease, SD standard deviation, SE standard error.

Change in CFS from baseline was assessed in 85 patients in the preservative-free latanoprost eye drop emulsion arm and 89 in the preserved latanoprost arm with baseline CFS ≥ 1. Preservative-free latanoprost eye drop emulsion demonstrated statistical superiority in reduction of OSD signs with mean CFS score improvements from baseline at Week 12 (1.4 [SD: 0.5] to 0.8 [0.5]) versus preserved latanoprost (1.3 [0.5] to 1.1 [0.6]); with a difference in LS mean changes of −0.3 ([SE: 0.1; p = 0.001]; Fig. 3A).

Change in OSD average symptom score from baseline was assessed in 105 patients in the preservative-free latanoprost eye drop emulsion arm and 108 patients in the preserved latanoprost arm with baseline OSD symptom score >0. At Week 12, LS mean improvement (SE) in OSD symptom score from baseline was numerically greater with preservative-free latanoprost eye drop emulsion (−0.3 [0.1]) than with preserved latanoprost (−0.2 [0.1]; p = 0.090). Mean (SD) OSD symptom score at baseline was 0.7 (0.5) in the preservative-free latanoprost eye drop emulsion arm and 0.7 (0.4) in the preserved latanoprost arm, decreasing to 0.4 (0.4) and 0.5 (0.5) at Week 12, respectively (Fig. 3B).

### Other efficacy endpoints

Treatment was reported as ‘satisfactory’ or ‘very satisfactory’ after 12 weeks by 98.4% (n = 188) of patients in the preservative-free latanoprost eye drop emulsion arm (including 52.4% [n = 100] who were very satisfied) and 90.5% (n = 172) of patients in the preserved latanoprost arm (with 36.3% [n = 69] who were very satisfied; nominal p < 0.001 [Supplementary Fig. 5]). Other secondary endpoints (TFBUT, conjunctival hyperaemia and slit lamp examination [MGD, conjunctiva chemosis, and lid and tear film debris]) were broadly similar between treatment arms (Supplementary Table 1).

### Safety

The proportion of patients with any AE was 18.1% (n = 35) in the preservative-free latanoprost eye drop emulsion arm and 21.8% (n = 42) in the preserved latanoprost arm (Table 2). Of these, 5.7% (n = 11) of patients in the preservative-free latanoprost eye drop emulsion arm compared with 10.9% (n = 21) of patients in the preserved latanoprost arm had AEs that were considered treatment-related. Ocular and conjunctival hyperaemia were the most common treatment-related AEs, occurring in 1.6% (n = 3) and 1.0% (n = 2) of patients treated with preservative-free latanoprost eye drop emulsion, respectively, and in 2.6% (n = 5) and 1.6% (n = 3) of patients treated with preserved latanoprost. Serious AEs occurred in one patient in the preservative-free latanoprost eye drop emulsion arm and in two patients in the preserved latanoprost arm, though none were reported as treatment-related. There were three treatment discontinuations due to AEs. One patient in the preservative-free latanoprost eye drop emulsion arm discontinued due to eye pain, eye pruritis and relapse of burning mouth syndrome. One patient in the preserved latanoprost arm discontinued due to eye irritation and ocular hyperaemia. One patient in the preservative-free latanoprost eye drop emulsion arm died due to acute heart failure that was considered by the investigator to be unrelated to the study drug. At Week 12, the mean (SD) change from baseline in BCDVA was −0.004 (0.05) with preservative-free latanoprost eye drop emulsion and −0.003 (0.05) with preserved latanoprost.

**Table 2:** Adverse events in patients included in the safety analysis.

	Preservative-free latanoprost eye drop emulsion (n = 193)^a^	Preserved latanoprost (n = 193)^a^
Any AE, n (%)	35 (18.1)	42 (21.8)
Ocular AEs, n (%)	20 (10.4)	26 (13.5)
Non-ocular AEs, n (%)	20 (10.4)	21 (10.9)
Infections and infestations	6 (3.1)	8 (4.1)
Musculoskeletal and connective tissue disorders	4 (2.1)	4 (2.1)
Nervous system disorders	3 (1.6)	1 (0.5)
Cardiac disorders	2 (1.0)	0
Gastrointestinal disorders	2 (1.0)	0
Respiratory, thoracic and mediastinal disorders	2 (1.0)	2 (1.0)
General disorders and administration site conditions	1 (0.5)	2 (1.0)
Injury, poisoning and procedural complications	1 (0.5)	1 (0.5)
Investigations^b^	1 (0.5)	2 (1.0)
Hepatobiliary disorders	0	1 (0.5)
Neoplasms	0	1 (0.5)
Surgical/medical procedures	0	1 (0.5)
SAEs, n (%)	1 (0.5)	2 (1.0)
Treatment-related AEs^c^ reported in ≥1% of patients, n (%)		
Any	11 (5.7)	21 (10.9)
Eye disorders	10 (5.2)	18 (9.3)
Ocular hyperaemia	3 (1.6)	5 (2.6)
Conjunctival hyperaemia	2 (1.0)	3 (1.6)
Erythema of eyelid	1 (0.5)	2 (1.0)
Eye pruritus	1 (0.5)	2 (1.0)
Swelling of eyelid	1 (0.5)	2 (1.0)
Abnormal sensation in eye	0	3 (1.6)
Eye irritation	0	2 (1.0)
Foreign body sensation in eye	0	2 (1.0)
General disorders and administration site conditions	0	3 (1.6)
Instillation site pain	0	3 (1.6)

*AE* adverse event, *SAE* serious adverse event.

^a^All patients who received a dose of study medication were included in the safety analysis. Statistical tests were not performed in the safety population.

^b^Investigations included increased blood cholesterol and increased body temperature.

^c^Treatment-related AEs were defined as any AE considered by the investigator to be related to the study drug, study procedures, or artificial tears; there were no AEs related to study procedures or artificial tears reported.

## Discussion

In this randomised Phase III study, the primary efficacy endpoint of non-inferiority of preservative-free latanoprost eye drop emulsion compared with preserved latanoprost was met. IOP was reduced by >8 mmHg from baseline in both treatment arms at Week 4 and IOP control was maintained throughout the 12-week study period. At Week 12 peak, preservative-free latanoprost eye drop emulsion demonstrated greater IOP-lowering efficacy versus preserved latanoprost with a treatment difference of −0.6 mmHg (nominal p = 0.023). While the clinical relevance of a treatment difference of <1 mmHg is still to be validated, research suggests that the risk of glaucoma progression may decrease by 10% with every 1 mmHg decrease in IOP [25]. Moreover, the applicability to patients with more advanced stages of glaucoma must also be considered [25].

At Week 12 trough, more patients experienced IOP reduction to ≤18 mmHg in the preservative-free latanoprost eye drop emulsion arm compared with patients in the preserved latanoprost arm (87.6% vs 79.3%, respectively [nominal p = 0.029]). Additionally, at Week 12 peak, 74.5% of patients experienced ≥30% IOP reduction (a target IOP reduction to slow moderate glaucoma progression [1]) compared with 64.0% of patients treated with preserved latanoprost (nominal p = 0.028). Taken together, these results indicate the IOP-lowering efficacy of preservative-free latanoprost eye drop emulsion is at least equivalent to that of preserved latanoprost in patients with OAG or OHT.

Effective management of OSD may improve glaucoma treatment adherence and IOP control, such that long-term vision is preserved and quality of life maintained [1, 10, 26]. However, OSD in patients with glaucoma is frequently unrecognised and untreated, but when specifically assessed, it has been reported in as many as 59% of patients based on symptoms and up to 78% based on signs [3–5]. In this study, 45.3% of patients had a baseline CFS score of ≥1. As the presence of OSD was not a requirement for study entry, the incidence rate of patients with corneal damage shows that even in patients receiving monotherapy, OSD may be present and should therefore be carefully assessed and adequately managed.

Cationic emulsions improve ocular surface health through stabilising the tear film by compensating for meibum deficiency at the tear film lipid layer interface [27, 28], and have demonstrated clinical efficacy in improving signs and symptoms in patients with DED [17, 18]. The cationic emulsion formulation of preservative-free latanoprost also appeared to confer clinical benefits to the ocular surface, as evidenced by the greater reduction in CFS score at Week 12 (mean change of −0.7 with preservative-free latanoprost eye drop emulsion vs −0.4 with preserved latanoprost [nominal p < 0.001]) and greater reduction in OSD symptom score at Week 12 versus preserved latanoprost (difference of −0.3 vs −0.2, respectively [nominal p = 0.09]), representing a clinically meaningful improvement as it is a > 50% change towards the next OSD grade (the threshold between grades of OSD is 0.5) [29]. OSD symptom score improvement in the preserved latanoprost arm at Week 12 compared with Week 4 was observed and may have been influenced by the variability in the assessment of OSD symptom scoring. Several studies have reported a connection between OSD management and improved IOP control. In a study of 10 patients with OAG and symptoms of OSD who switched from topical anti-glaucoma medications to preservative-free equivalents, had allergenic treatments or those causing side effects, or switched to another therapeutic class with the same efficacy but a more tolerable safety profile, and treatment of OSD reported improvement in ocular surface health in all patients. The switches were also associated with a decreased or stabilised IOP; mean IOP significantly decreased from 23.8 ± 10.0 mmHg to 15.2 ± 4.8 mmHg (−36.2%; p < 0.001) [26]. In a prospective interventional study of 19 patients with OAG or angle-closure glaucoma and OSD, 6 months of OSD treatment resulted in an IOP reduction of −1.6 mmHg compared with baseline (p = 0.051) [5]. A potential mechanistic explanation for these findings is the reduction in inflammatory stimuli that affect the trabecular meshwork and thus lowering IOP [5].

The improvement in CFS at Week 12 with preservative-free latanoprost eye drop emulsion versus preserved latanoprost may indicate better tolerability of the former, as discomfort caused by OSD may contribute to a lack of adherence to glaucoma medication and thus disease progression (including poorly controlled IOP and increased visual deterioration) [10, 30, 31]. Furthermore, patient satisfaction is known to have a significant positive direct influence on medication adherence [30]. Patient satisfaction scores were high across treatment arms; 98.4% of patients in the preservative-free latanoprost eye drop emulsion arm and 90.5% of patients in the preserved latanoprost arm reported their treatment as ‘satisfactory’ or ‘very satisfactory’. Improvements in OSD signs and symptoms, and higher patient satisfaction compared with preserved latanoprost in this study, suggest there may be a potential for preservative-free latanoprost eye drop emulsion to improve adherence and thus overall glaucoma management [32]; however, longer-term studies would be required.

While both therapies in this study were well tolerated, a lower percentage of treatment-related AEs were observed in the preservative-free latanoprost eye drop emulsion arm (5.7%) compared with the preserved latanoprost arm (10.9%). Consistent with the known safety profile of latanoprost [33], the most common treatment-related AEs were ocular AEs including conjunctival hyperaemia, though this was less common with preservative-free latanoprost eye drop emulsion than with preserved latanoprost.

The results of this Phase III study align with preliminary Phase II data [24], and confirm the IOP-lowering efficacy of preservative-free latanoprost eyedrop emulsion in patients with OAG and/or OHT, with or without OSD. Key secondary endpoints of CFS score and OSD symptoms indicated benefits of preservative-free latanoprost eye drop emulsion in improving OSD signs and symptoms. While both treatment options demonstrated a good safety profile, preservative-free latanoprost eye drop emulsion induced fewer treatment-related AEs than preserved latanoprost, and showed a higher patient satisfaction rate.

## Conclusions

This Phase III trial demonstrates a dual benefit with preservative-free latanoprost eye drop emulsion of comparable IOP-lowering versus preserved latanoprost, and improvements to the ocular surface. Preservative-free latanoprost eye drop emulsion represents an innovative evolution for latanoprost monotherapy and a valuable addition to the glaucoma treatment armamentarium.

Supplemental material is available at Eye’s website.

## Summary

### What was known before

- Glaucoma management aims to preserve vision and quality of life by reducing intraocular pressure (IOP).
- Patients with glaucoma often have concomitant ocular surface disease, which can impact glaucoma treatment adherence and the effectiveness of IOP-lowering medications.

### What this study adds

- This study evidences the dual benefit of preservative-free latanoprost eye drop emulsion, with non-inferior IOP-lowering efficacy compared with preserved latanoprost, and improvements to the ocular surface.
- With a cationic emulsion formulation, preservative-free latanoprost eye drop emulsion represents a valuable addition to the glaucoma treatment armamentarium.

## Supplementary information

Supplementary information

## Data Availability

The data that support the findings of this study may be requested from the corresponding author.
