# Supplementary material for: A phase III study comparing preservative-free latanoprost eye drop emulsion with preserved latanoprost in open-angle glaucoma or ocular hypertension
Source: Eye (Lond). 2025 Feb 25;39(8):1599–607. doi: 10.1038/s41433-025-03646-z (PMC12089586; doi:10.1038/s41433-025-03646-z)

# Supplementary materials for:

# A Phase III study comparing preservative-free latanoprost eye drop emulsion with preserved latanoprost in open-angle glaucoma or ocular hypertension

Christophe Baudouin, Ingeborg Stalmans, Rupert Bourne, Jose Manuel Larrosa, Stefanie Schmickler, Aleksey Seleznev and Francesco Oddone, on behalf of the Phase III study group

## Supplementary Statistical Information

Analysis of the primary efficacy endpoint was performed on the FAS population using a MMRM model on observed cases collected up to Week 12 [1]. No imputation of missing data was required. A separate MMRM model was used for IOP at each timepoint. The model included treatment, visit, treatment-by-visit interaction as fixed effects, baseline IOP at the respective timepoint and country as covariates. Within-subject errors will be modelled using an unstructured (UN) covariance matrix. Where the UN model failed to converge, the first-order auto-regression model and the variance components model were fitted sequentially until the convergence criteria are met.

A similar MMRM to primary endpoint analysis was performed mean diurnal IOP in period 1. The model included treatment, visit, and treatment-by-visit interaction as fixed effects, baseline diurnal IOP and country as covariates. Estimates of treatment effects were based on the least square means (LS mean). The LS mean, difference of the LS mean between two groups, and the corresponding 95% CI was provided at each visit.

For the binary endpoints, number of patients who achieved the criteria, response rate (%), risk difference and the corresponding 95% confidence interval, and p-value (Chi-squared test) were provided.

**Reference**

1. Laird NM, Ware JH. Random-Effects Models for Longitudinal Data. Biometrics 1982;38:963–74.

## Supplementary Table 1. TFBUT, conjunctival hyperaemia and slit lamp examination in the efficacy analysis

| **Endpoint** | **Analysis visit** | **Statistics (Scores)** | **Preservative-free latanoprost eye drop emulsion (n=192)** | **Preserved latanoprost (n=192)** |
| --- | --- | --- | --- | --- |
| TFBUT | Baseline | n | 123 | 136 |
|  |  | Mean (SD) | 5.88 (2.71) | 6.14 (2.67) |
|  | Week 4 | n | 121 | 134 |
|  |  | Mean (SD) | 6.49 (2.92) | 6.94 (3.42) |
|  | Week 12 | n | 119 | 134 |
|  |  | Mean (SD) | 6.67 (3.14) | 7.03 (3.59) |
| Conjunctival hyperaemia score | Baseline | n | 191 | 191 |
|  |  | Mean (SD) | 1.38 (0.58) | 1.42 (0.63) |
|  | Week 4 | n | 189 | 189 |
|  |  | Mean (SD) | 1.38 (0.59) | 1.46 (0.66) |
|  | Week 12 | n | 190 | 193 |
|  |  | Mean (SD) | 1.34 (0.55) | 1.42 (0.66) |
| Slit lamp examination |  | | | |
| Meibomian glands | Baseline | n | 192 | 192 |
|  |  | Mean (SD) | 0.4 (0.65) | 0.4 (0.66) |
|  | Week 4 | n | 189 | 189 |
|  |  | Mean (SD) | 0.4 (0.65) | 0.3 (0.60) |
|  | Week 12 | n | 188 | 190 |
|  |  | Mean (SD) | 0.4 (0.60) | 0.3 (0.59) |
| Conjunctiva chemosis | Baseline | n | 192 | 192 |
|  |  | Mean (SD) | 0.1 (0.34) | 0.2 (0.40) |
|  | Week 4 | n | 189 | 189 |
|  |  | Mean (SD) | 0.1 (0.25) | 0.1 (0.37) |
|  | Week 12 | n | 188 | 190 |
|  |  | Mean (SD) | 0.0 (0.21) | 0.1 (0.24) |
| Lid and lid margin erythema | Baseline | n | 192 | 192 |
|  |  | Mean (SD) | 0.3 (0.55) | 0.4 (0.58) |
|  | Week 4 | n | 189 | 189 |
|  |  | Mean (SD) | 0.3 (0.53) | 0.3 (0.52) |
|  | Week 12 | n | 188 | 190 |
|  |  | Mean (SD) | 0.3 (0.51) | 0.3 (0.48) |
| Tear film debris | Baseline | n | 192 | 192 |
|  |  | Mean (SD) | 0.2 (0.48) | 0.2 (0.47) |
|  | Week 4 | n | 189 | 189 |
|  |  | Mean (SD) | 0.2 (0.40) | 0.2 (0.45) |
|  | Week 12 | n | 188 | 190 |
|  |  | Mean (SD) | 0.2 (0.41) | 0.1 (0.35) |

SD, standard deviation; TFBUT, tear-film breakup time.

Supplementary Figure 1. Study design
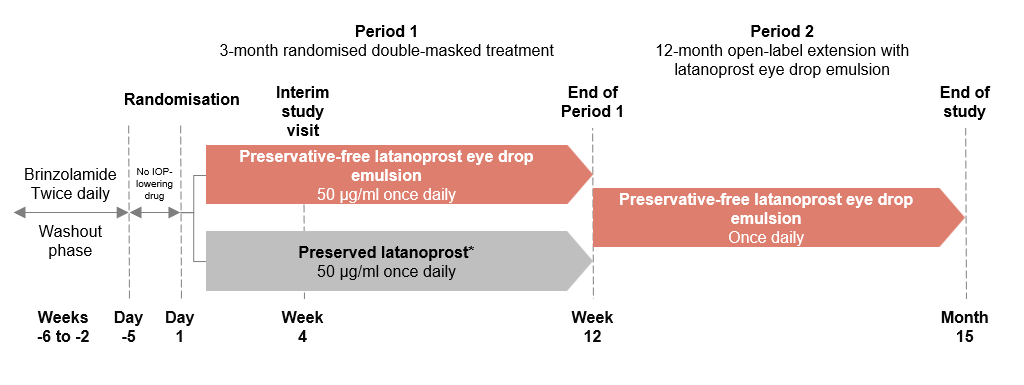


*Preserved latanoprost was requested as a study reference drug by the European Medicines Agency.

IOP, intraocular pressure.

## Supplementary Figure 2. Patient disposition


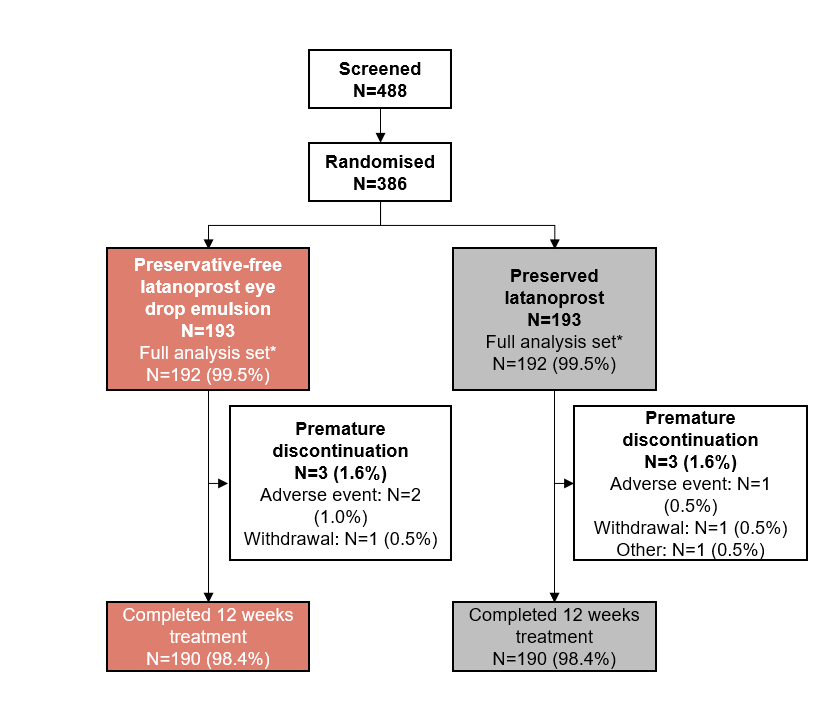
*All randomised patients received at least one dose of study medication and were included in the safety population (N=386, 193 in each treatment arm); 384 patients (192 in each treatment arm) had at least one post-baseline IOP measurement at both peak and trough times and were included in the full analysis set evaluated for efficacy.
IOP, intraocular pressure.

## Supplementary Figure 3. Baseline CFS and OSD average symptom score


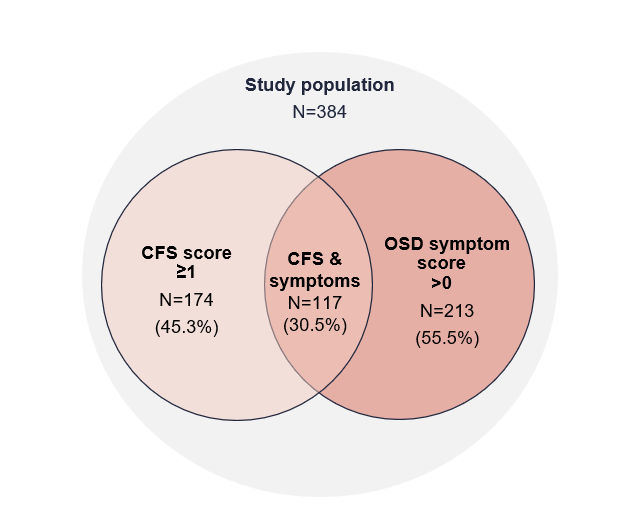
CFS, corneal fluorescein staining; OSD, ocular surface disease.

## Supplementary Figure 4. Patients with IOP response at Week 12 (A) peak and (B) trough in the efficacy population

**A**
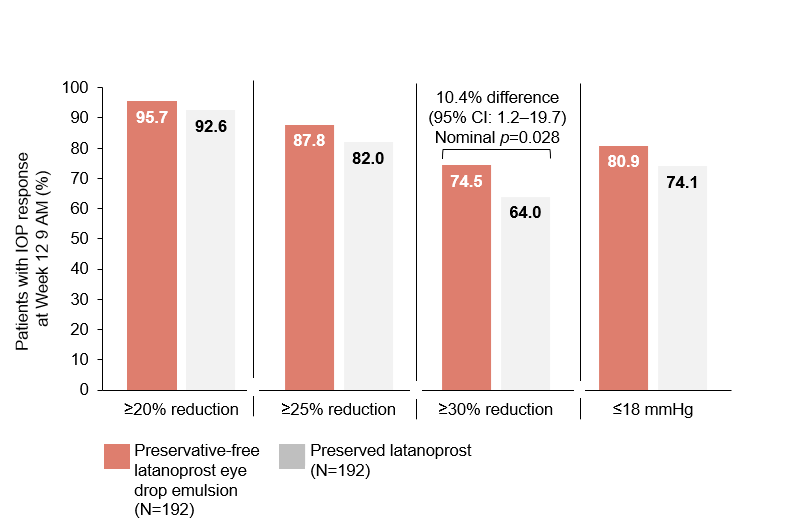


**B**

**
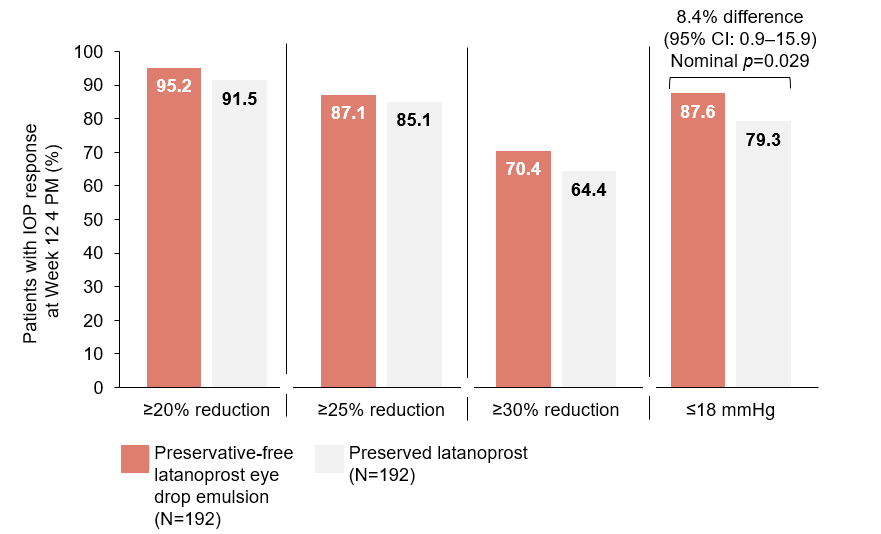
**

CI, confidence interval; IOP, intraocular pressure.

Supplementary Figure 5. Patient Global Rating of Treatment after 12 weeks in the efficacy analysis
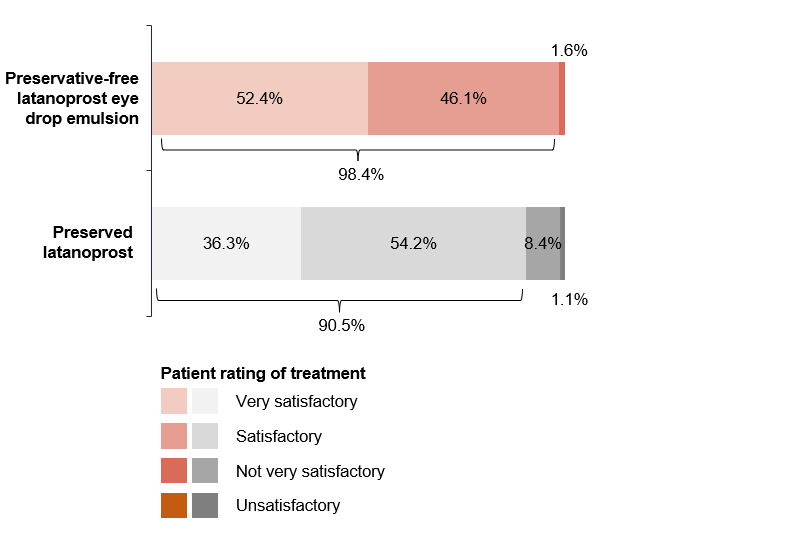

Supplement: Supplementary file 1 — Supplementary information [file 41433_2025_3646_MOESM1_ESM.docx]
